# The Adult Cystic Fibrosis Airway Microbiota Is Stable over Time and Infection Type, and Highly Resilient to Antibiotic Treatment of Exacerbations

**DOI:** 10.1371/journal.pone.0045001

**Published:** 2012-09-26

**Authors:** Anthony A. Fodor, Erich R. Klem, Deirdre F. Gilpin, J. Stuart Elborn, Richard C. Boucher, Michael M. Tunney, Matthew C. Wolfgang

**Affiliations:** 1 Department of Bioinformatics and Genomics, University of North Carolina at Charlotte, Charlotte, North Carolina, United States of America; 2 Cystic Fibrosis/Pulmonary Research and Treatment Center, University of North Carolina at Chapel Hill, Chapel Hill, North Carolina, United States of America; 3 CF and Airway Microbiology Research Group, Queen's University Belfast, Belfast, United Kingdom; 4 School of Pharmacy, Queen's University Belfast, Belfast, United Kingdom; 5 Center for Infection and Immunity, School of Medicine, Dentistry and Biomedical Sciences, Queen's University Belfast, Belfast, United Kingdom; 6 Department of Microbiology and Immunology, University of North Carolina at Chapel Hill, Chapel Hill, North Carolina, United States of America; Univeristy of California Berkeley, United States of America

## Abstract

Cystic fibrosis (CF) is characterized by defective mucociliary clearance and chronic airway infection by a complex microbiota. Infection, persistent inflammation and periodic episodes of acute pulmonary exacerbation contribute to an irreversible decline in CF lung function. While the factors leading to acute exacerbations are poorly understood, antibiotic treatment can temporarily resolve pulmonary symptoms and partially restore lung function. Previous studies indicated that exacerbations may be associated with changes in microbial densities and the acquisition of new microbial species. Given the complexity of the CF microbiota, we applied massively parallel pyrosequencing to identify changes in airway microbial community structure in 23 adult CF patients during acute pulmonary exacerbation, after antibiotic treatment and during periods of stable disease. Over 350,000 sequences were generated, representing nearly 170 distinct microbial taxa. Approximately 60% of sequences obtained were from the recognized CF pathogens *Pseudomonas* and *Burkholderia*, which were detected in largely non-overlapping patient subsets. In contrast, other taxa including *Prevotella*, *Streptococcus*, *Rothia* and *Veillonella* were abundant in nearly all patient samples. Although antibiotic treatment was associated with a small decrease in species richness, there was minimal change in overall microbial community structure. Furthermore, microbial community composition was highly similar in patients during an exacerbation and when clinically stable, suggesting that exacerbations may represent intrapulmonary spread of infection rather than a change in microbial community composition. Mouthwash samples, obtained from a subset of patients, showed a nearly identical distribution of taxa as expectorated sputum, indicating that aspiration may contribute to colonization of the lower airways. Finally, we observed a strong correlation between low species richness and poor lung function. Taken together, these results indicate that the adult CF lung microbiome is largely stable through periods of exacerbation and antibiotic treatment and that short-term compositional changes in the airway microbiota do not account for CF pulmonary exacerbations.

## Introduction

Cystic fibrosis (CF) is a hereditary disease characterized by defective mucociliary clearance and airway obstruction by dehydrated, mucopurulent secretions [1]. The leading cause of morbidity and mortality in individuals with CF is respiratory failure associated with persistent airway infection and inflammation [2]–[4]. Traditional aerobic culture-based studies, of spontaneous expectorated sputa, indicate that CF airway infection involves a relatively small collection of opportunistic pathogens that are typically acquired in a temporal succession, beginning early in life with *Haemophilus influen*zae and *Staphylococcus aureus* and culminating in chronic infections dominated by *Pseudomonas aeruginosa* or *Burkholderia cepacia* complex species [5]. Additional opportunistic pathogens, including *Stenotrophomonas maltophilia*, *Achromobacter xylosoxidans* and nontuberculous *mycobacterium* have been recovered from adult patients with increasing frequency [6]. The observation that the mucous obstructing CF airways is hypoxic [7] has led to multiple studies in which CF respiratory specimens have been analyzed under strict anaerobic culture conditions [8]–[10]. These studies have shown that anaerobic bacterial species are also present within CF airways in high numbers. The spectrum of facultative and obligate anaerobic species recovered from CF specimens frequently includes members of the genera *Streptococcus*, *Prevotella*, *Actinomyces* and *Veillonella*. The use of culture independent molecular detection methods has greatly expanded our understanding of the complexity of CF airway disease. High throughput sequencing efforts indicate that the CF microbiome consists of more than 60 different bacterial genera, while interrogation of bacterial 16S ribosomal RNA (rRNA) gene-based phylogenetic microarrays has placed the estimate at as many as 43 different bacterial phyla and over 2,000 different taxa [11]–[13].

**Table 1:** Antibiotic regimens used to treat acute pulmonary exacerbations in this study^1^.

Antibiotic regimen	Number of exacerbations treated
Tobramycin, Ceftazidime	8
Tobramycin, Piperacillin/Tazobactam	6
Tobramycin, Meropenem	2
Tobramycin, Ciprofloxacin	1
Tobramycin, Temocillin	1
Tobramycin, Ciprofloxacin, Colomycin	1
Tobramycin, Piperacillin/Tazobactam, Meropenem	1
Temocillin, Colomycin	2
Temocillin, Meropenem	1
Piperacillin/Tazobactam, Colomycin, Ciprofloxacin	1
Amikacin, Meropenem	1
Amikacin, Clindamycin, Moxifloxacin, Clarithromycin	1

1 Table indicates antibiotic regimens used to treat each of the 26 exacerbations that occurred during the period of study. Of the 23 patients enrolled, three patients experienced two separate exacerbations. For these three patients, the first and second exacerbations were treated with different antibiotic combinations.

**Figure 1:**
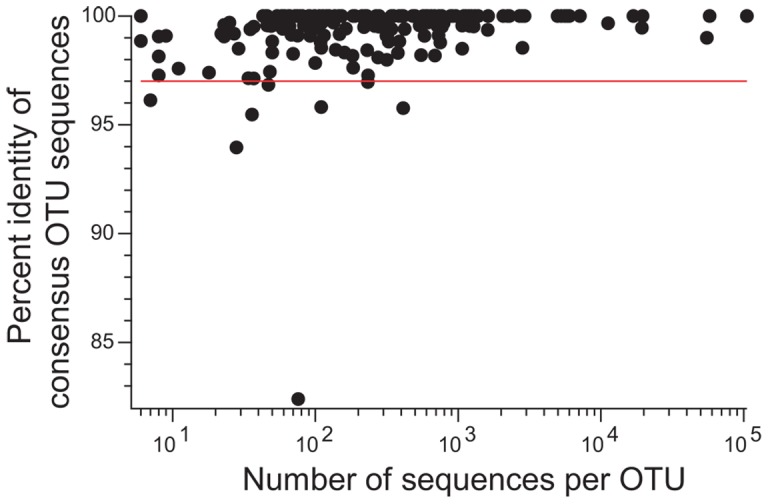
The CF microbiome consists primarily of previously identified bacterial taxa. Each consensus OTU identified within the CF sample set was aligned to version 104 of the SILVA database of bacterial 16S rRNA gene sequences using the program align.seqs in the software package Mothur as described in Methods. The percent identity of the resulting global alignment is shown as a function of the number of sequences in each OTU. The red line represents 97% identity.

Despite the complexity and variety of microbial organisms that can reside in the airways of CF patients, individuals can exhibit relatively extended periods of stable disease symptoms. These stable periods are punctuated by episodes of acute pulmonary exacerbation. While the definition of a pulmonary exacerbation is controversial, it generally involves a rapid decline in pulmonary function and worsening of respiratory symptoms, requiring antibiotic treatment and hospitalization [14]. Patients who experience more frequent exacerbations have increased morbidity and mortality [15], [16]. Furthermore, recent studies have demonstrated an association between exacerbation frequency and the long-term rate of lung function decline [16], [17]. The factors contributing to acute pulmonary exacerbation are poorly understood. Infection by respiratory viruses may be a precipitating event and some studies suggest that up to a third of CF exacerbations are associated with viral infection [18]. Other recent studies have shown a correlation between acute exacerbation and airway colonization by bacteria belonging to the *Streptococcus milleri* group [19]–[21]. Furthermore, it has been proposed that species of the genus *Prevotella* may play a role in exacerbation; however, direct evidence for this association is lacking [22], [23]. Interestingly, both *Streptococcus milleri* and *Prevotella* species are associated with endogenous anaerobic infections in multiple sites including the respiratory tract [20], [24]–[26].

**Figure 2:**
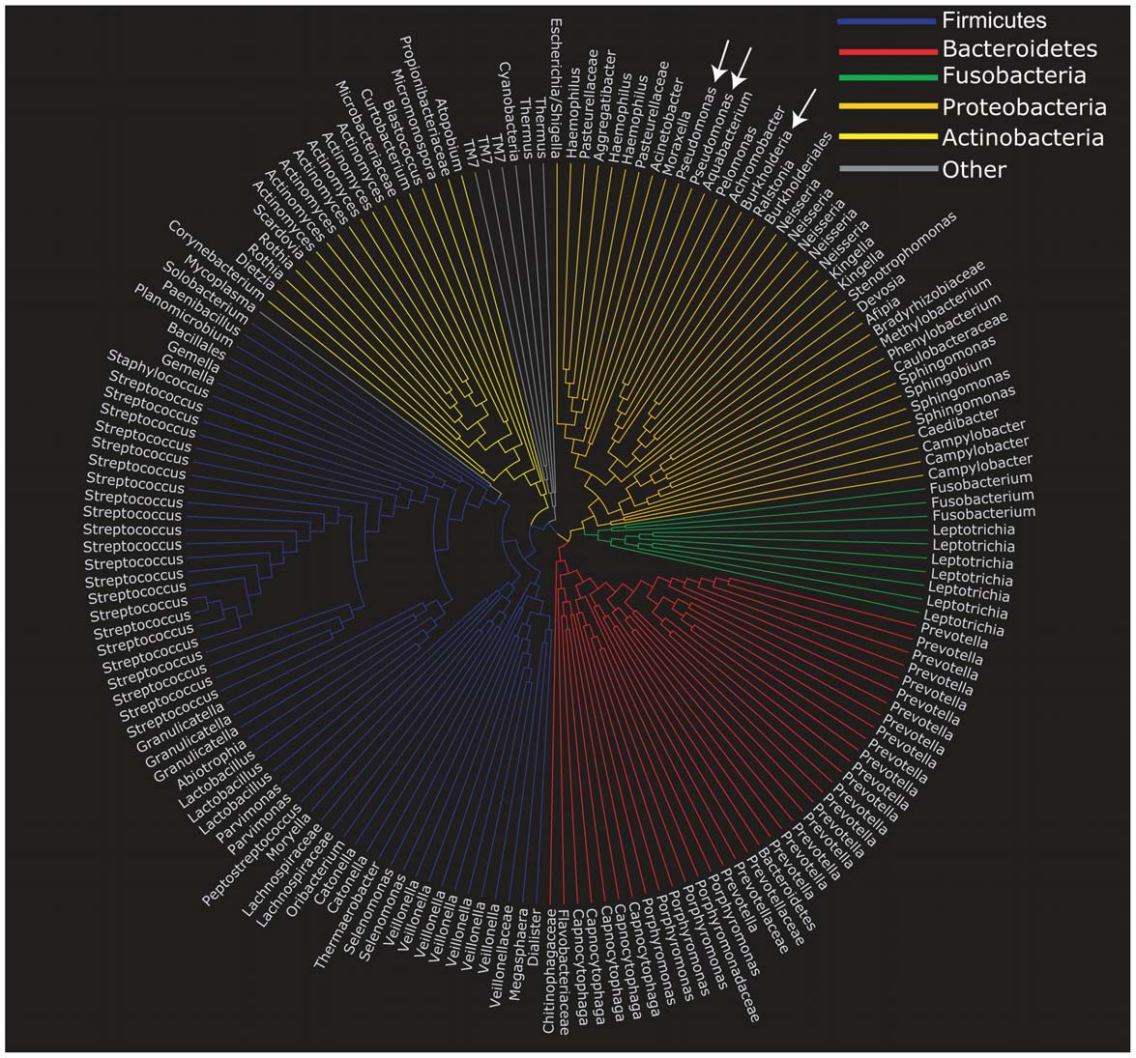
CF is a polymicrobial disease. Phylogenetic tree of the 169 OTUs identified in the CF sputum dataset. Tree construction was achieved by mapping consensus sequences from each OTU to the SILVA reference tree (see Methods). Each leaf of the tree represents a consensus OTU labeled with the most closely related genus assigned by the RDP classifier.

Previous studies utilizing aerobic and strict anaerobic bacterial culture techniques indicate that polymicrobial CF pulmonary infection persists through multiple rounds of antibiotic treatment for acute exacerbations [8], [10]. The results of these studies raise the question as to whether acute exacerbations are caused by an intensification of pre-existing bacterial species, emergence of new species, or intrapulmonary spread of infection.

**Figure 3:**
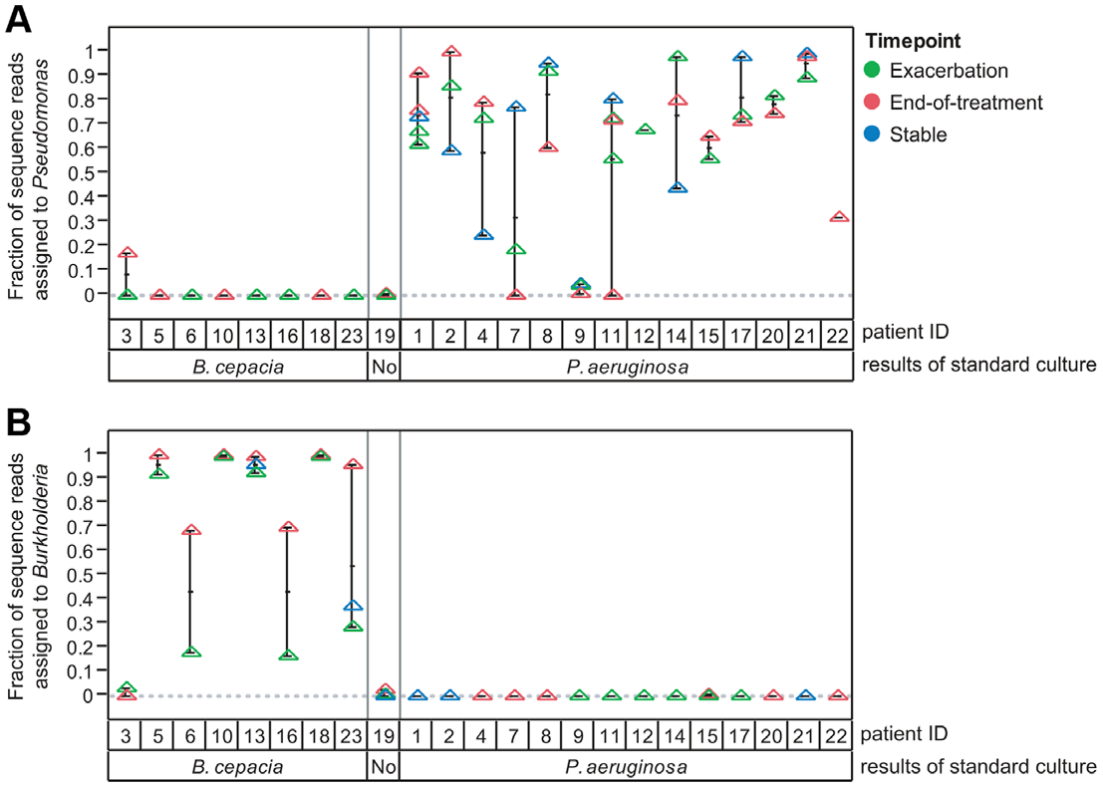
There is broad agreement between qualitative bacterial culture results and 454 pyrosequencing for dominant CF pathogens. The fraction of sequence assigned to the genus (a) *Pseudomonas* or (b) *Burkholderia* are plotted for each patient and sample timepoint as a function of the patient' s reported culture status for the recognized CF pathogens *P. aeruginosa* and *B. cepacia* complex species. Patient samples are color-coded by timepoint (green, onset of exacerbation; red, end-of-treatment for exacerbation with intravenous antibiotics; blue, clinically stable interval). Patient 19 was culture negative for both *P. aeruginosa* and *B. cepacia*.

The application of high-throughput sequencing provides a detailed view of the complex microbial communities that exist in the CF lung [11]–[13]. In contrast to healthy individuals, CF patients are subjected to repeated rounds of antibiotics to control chronic and recurrent respiratory infection. High-throughput sequencing provides a means to characterize the effects of antibiotics on airway microbial communities that have likely evolved and adapted in response to prior antibiotic treatment. Further, comparison of the microbiome of CF patients experiencing an acute exacerbation to both post antibiotic and stable intervals may provide insight into the mechanistic basis of acute exacerbation.

**Figure 4:**
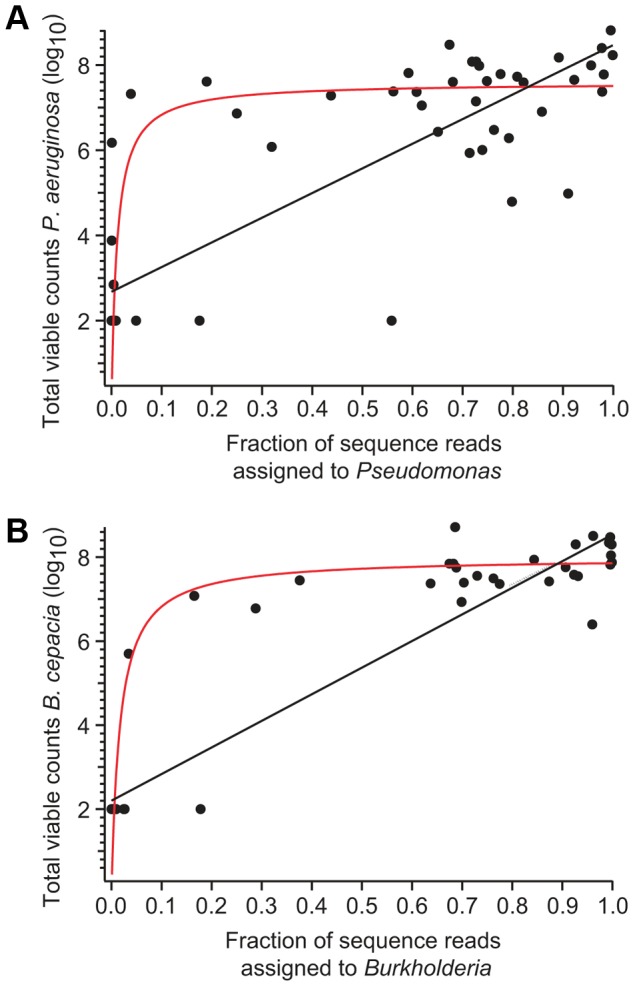
Total viable counts by culture show significant but non-linear agreement with relative sequence abundance. TVC for (a) *P. aeruginosa* and (b) *B. cepacia* complex species plotted against the fraction of sequences assigned to the corresponding genera in each sputum sample. Black lines represent linear regression by least squares fitting. Values for *Pseudomonas* (r^2^ = 0.71, p<0.001) and *Burkholderia* (r^2^ = 0.86, p<0.001) indicate a significant correlation. Red lines are intended to illustrate a potential non-linear relationship and are based on the two-parameter Michaelis-Menten function with arbitrarily selected parameters.

In this study, we collected spontaneous expectorated sputum from 23 adult CF patients at the beginning of an acute exacerbation and after one course of antibiotic treatment for the exacerbation. Further, we collected sputum from 13 of the 23 patients when they were clinically stable and mouthwash samples from 9 of the patients at multiple timepoints. The sputum samples from this patient cohort were previously analyzed for bacterial content using both culture and culture-independent techniques [8]. Specifically, the results of quantitative aerobic and anaerobic culture and quantitative polymerase chain reaction (qPCR) to determine total bacterial 16S rRNA gene copies were published. Here, we report further analyses of the samples using 454 pyrosequencing to assess, at high resolution, changes in microbial community structure and species richness at the onset of exacerbation, following antibiotic treatment and during intervals of stable disease. Additionally, we assessed whether pulmonary function correlated with species richness and microbial densities in our well-characterized patient cohort.

**Figure 5:**
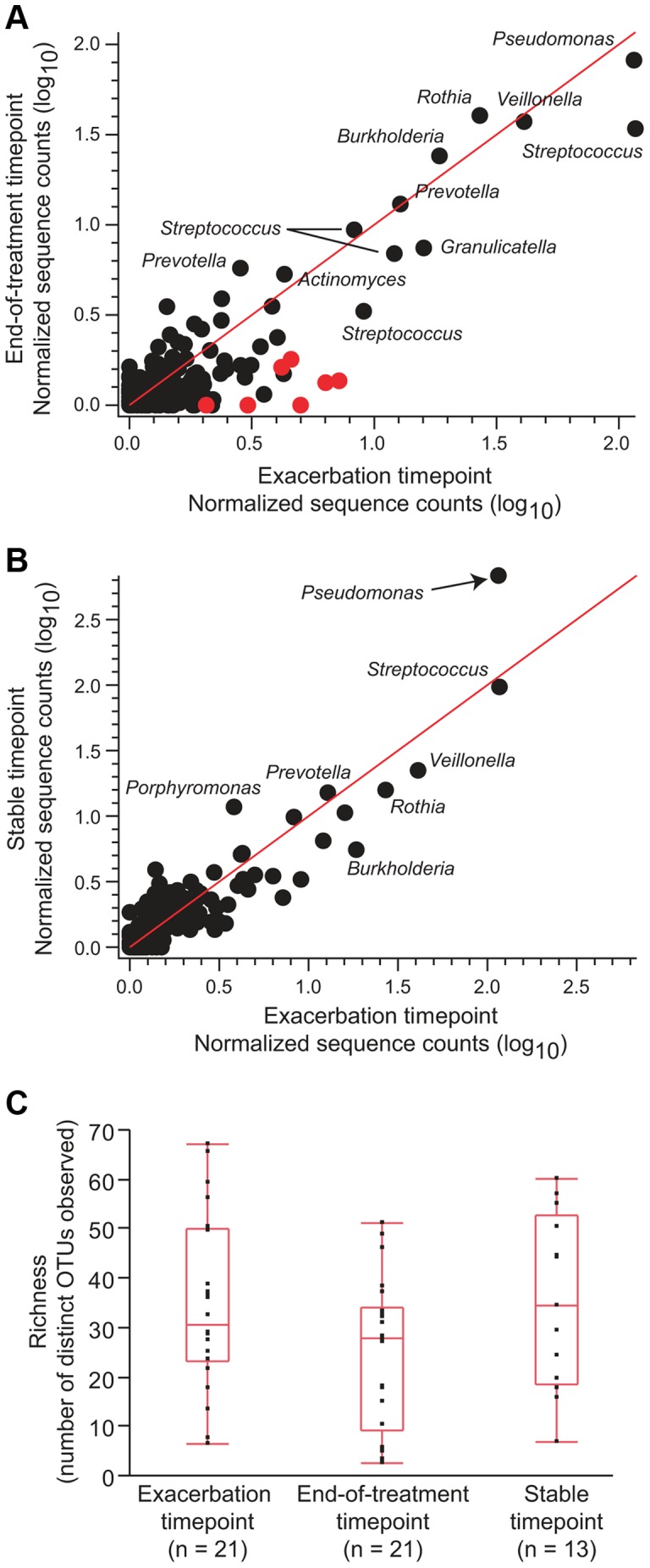
Abundance taxa are highly stable during exacerbation and in response to antibiotic treatment. To determine whether the relative abundance of specific taxa changed during exacerbation or following antibiotic treatment, the normalized average sequence abundance for all detected OTUs was compared (a) between exacerbation (n = 22) and end-of-treatment timepoints (n = 22) and (b) between the exacerbation (n = 22) and stable timepoints (n = 13). For each taxon, normalized average sequence abundance values are plotted as a logarithm to the base 10 (log_10_). Red circles indicate taxa that had significantly lower normalized average sequence abundance following antibiotic treatment at a 10% false discovery rate. (c) Comparison of overall microbial richness at all three sampling timepoints indicates a slight, but transient decrease following antibiotic treatment. By pairwise t-tests, comparisons of richness between exacerbation and end-of-treatment (p = 0.06, n = 21), exacerbation and stable (p = 0.87, n = 13) and stable and end-of-treatment (p = 0.076, n = 13) timepoints all fail to reach statistical significance at a p≤0.05 threshold.

## Results

### Antibiotic treatment of acute exacerbations is associated with temporary improvement of clinical indicators of pulmonary infection and function

Spontaneous expectorated sputum samples were collected from 23 adult CF patients at the onset of a clinically diagnosed exacerbation and following the completion of treatment with intravenous antibiotics. Enrollment and characterization of the patient cohort, was described as part of a previous clinical study [8]. Three of the patients were treated twice for an exacerbation resulting in a total of 26-matched exacerbation/end-of-treatment sample pairs. A wide range of antibiotic regimens was employed to treat exacerbations (Table 1). The antibiotic combinations used in this study were primarily selected to target known pathogens such as *P. aeruginosa* and *B. cepacia*; however, all of the regimens are expected to have broad-spectrum activity against both Gram-positive and Gram-negative bacteria. In addition, the majority of patients also received oral azithromycin or inhaled colistin or tobramycin as chronic maintenance therapy. Sputum was also collected from 13 of these patients when clinically stable with 7 samples collected prior to exacerbation, 5 samples collected post-exacerbation, and one sample collected between treatment of two separate exacerbations.

**Figure 6:**
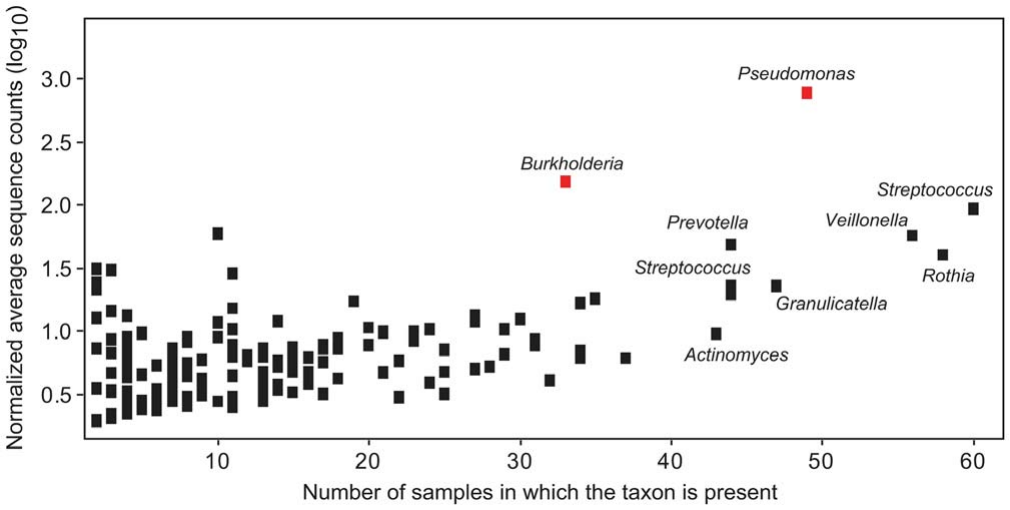
Prevalent taxa are also abundant taxa. Plot showing the log transformed (log_10_) average normalized sequence counts for each taxon compared to the number of samples in which the taxon is present. Averaged values only include samples in which the taxon is present. Only taxa present in two or more samples (155 OTUs) are plotted. Raw data used to generate used for this analysis are available in Table S7. Red symbols indicate recognized dominant CF pathogens.

Following antibiotic treatment for exacerbation, all patients showed clinical improvement; lung function, measured as forced expiratory volume (liters) in 1 second (FEV_1_), increased significantly and white cell count (WCC) and serum levels of C-reactive protein (CRP) decreased significantly [8]. Antibiotic treatment was associated with an average decrease (less than one log) in both total viable bacterial counts and reduced bacterial 16S rRNA gene copies (as measured by qPCR) in expectorated sputum [8]. For the 13 patients with an additional sputum sample obtained during stable disease, there was no difference in clinical parameters (FEV_1_, CRP, WCC) and bacterial density between the start of an exacerbation and stable timepoints [8].

**Figure 7:**
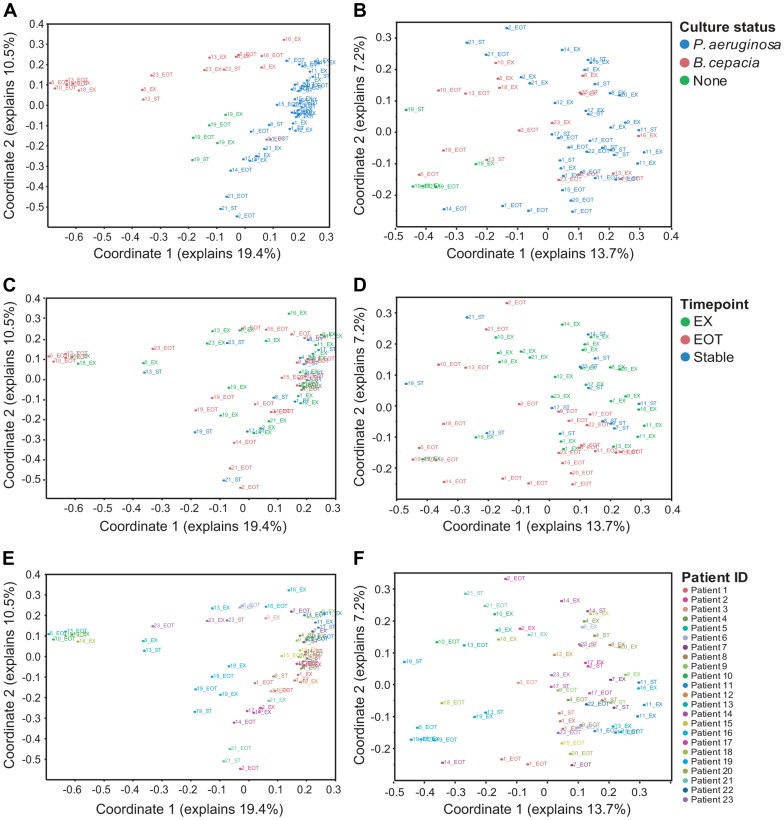
Sequence signatures discriminate between infection types, timepoint and individual patient. A principal coordinates analysis (PCoA) of the sputum sample sequence data was performed using Bray-Curtis distance. (a, b) PCoA with samples color-coded by patient' s reported culture status for the recognized CF pathogens *P. aeruginosa* (blue), *B. cepacia* complex species (red) or those that were culture negative for both (green). (c, d) PCoA in which samples are color-coded by timepoint; onset of acute exacerbation (EX, green), end-of-treatment with antibiotics (EOT, red) or when clinically stable (blue). (e, f) PCoA with samples color-coded by individual patient ID. Each sample is indicated by a symbol labeled with patient ID and timepoint (EX, onset of exacerbation; EOT, end-of-treatment with intravenous antibiotics; ST, clinically stable interval). (a, c, e) PCoA includes all taxa that had at least 10 sequences in the dataset. (b, d, f) PCoA with all *Pseudomonas* and *Burkholderia* sequences removed from the dataset. Relative abundance data (Table S3) for each OTU were log-transformed and normalized before Bray-Curtis dissimilarities were calculated and analyzed using the PCoA algorithm in the program Mothur as described in the Methods.

**Table 2:** P-values derived from PCoA for comparisons between individuals and patient groups defined by culture status.

	P-values			
	All sequences		*Pseudomonas* and *Burkholderia* sequences excluded	
Null hypothesis	Coordinate 1	Coordinate 2	Coordinate 1	Coordinate 2
1Patients with *Burkholderia* (n = 8) and *Pseudomonas* (n = 14) cluster together.	0.0002	0.008	0.10	0.71
2Samples from individual patients with *Pseudomonas* (n = 14) cluster together (40 timepoints).	0.06	0.003	0.02	0.08
2Samples from individual patients with *Burkholderia* (n = 8) cluster together (18 timepoints).	0.04	0.13	0.11	0.64
2Samples from individual patients (n = 23) cluster together across time.	0.0003	0.0004	0.001	0.15

1 The average value for each coordinate of the PCoA was taken for each patient (collapsing across all timepoints) and the Wilcoxon test was used to evaluate the hypothesis that the distribution of the resultant average coordinates was the same for *Pseudomonas*- and *Burkholderia-*positive patients.

2 To evaluate whether patients from the same culture status group clustered together across time, we used the Krusall-Wallis test with a factor of patient ID in which samples from different timepoints were treated as repeated samples for each patient. Because patients who were culture positive for *Pseudomonas* and *Burkholderia* clustered separately, we performed the analysis on each patient group as well as a combined analysis on all patients (*Pseudomonas*-positive, n = 14; *Burkholderia*-positive, n = 8; culture negative, n = 1).

### Pyrosequencing results are consistent with conventional culture for dominant pathogens and provide a high-resolution view of CF microbial community structure

To determine whether changes in CF airway microbial community structure are associated with exacerbation and antibiotic treatment, total microbial community DNA was isolated from expectorated sputum samples and subjected to 454 pyrosequencing targeting the V1–V2 region of the bacterial 16S rRNA gene. After quality control filtering (see Methods), the average number of sequences per sample was approximately 4,300 and all samples produced a minimum of 2,292 sequences (Table S1). Using version 2 of AbundantOTU [27], we identified a total of 169 non-chimeric microbial Operational Taxonomic Units (OTUs) at 97% identity in the CF sputum sample set (Tables S2–S5). A consensus sequence was generated for each OTU and compared to the SILVA rRNA Database of full-length 16S rRNA gene sequences [28]. The majority of consensus OTU sequences (77%) matched a reference sequence at 99% identity or greater (Figure 1). Only two OTUs shared less than 95% identity with a previously deposited reference sequence, indicating that nearly all of the taxa encountered in our CF patient cohort were previously detected in other sequencing projects. Furthermore, the high level of sequence identify between the OTU sequences generated here and full-length 16S rRNA reference sequences demonstrates a low overall rate of sequencing error and that the quality control pipeline used was sufficient for removing sequencing artifacts.

**Table 3:** P-values derived from PCoA for comparisons between sampling timepoints.

	P-values^1^			
	All sequences		*Pseudomonas* and *Burkholderia* sequences excluded	
Null hypothesis	Coordinate 1	Coordinate 2	Coordinate 1	Coordinate 2
Exacerbation timepoints and end of treatment timepoints have the same distribution (n = 21)	3.15×10−5	0.01	9.54×10−6	0.002
Exacerbation timepoints and stable timepoints have the same distribution (n = 13)	0.22	0.38	0.38	0.8926
End of treatment timepoints and stable timepoints have the same distribution (n = 13)	0.19	0.946	0.02	0.11

1 All P-values are the result of the paired t-tests.

A phylogentic tree constructed from the consensus OTU sequences (Figure 2) shows that the airway microbiota of our CF patient cohort includes taxa from multiple phyla including Bacteroidetes, Firmicutes, Proteobacteria, Fusobacteria, Actinobacteria and TM7. These results are consistent with previous studies that have characterized CF as a polymicrobial disease [8], [12], [13], [29]–[33]. The majority of sputum derived 16S rRNA gene sequences were assigned to two OTUs identified as *Pseudomonas* (38.5% of sequences) and a single OTU matching the genus *Burkholderia* (21.1% of sequences) (Table S2). These results are consistent with the fact that *Pseudomonas aeruginosa* and *Burkholderia cepacia* complex species are regarded as the dominant pathogens in adult CF populations and that all but one of the patients enrolled in this study were culture positive for at least one of these organisms [5], [8]. In contrast to the *Pseudomonas* and *Burkholderia* sequences, multiple distinct OTUs were assigned to other bacteria genera frequently associated with the oral microbiota, including *Streptococcus*, *Prevotella* and *Veillonella* (Figure 2, Table S2) [34], indicating greater diversity within these groups and presumably the presence of multiple distinct species or subspecies.

**Figure 8:**
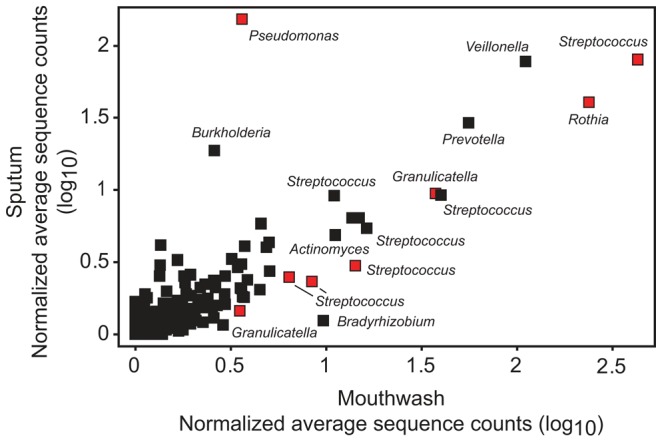
Mouthwash and sputum samples have a highly similar distribution of taxa. To determine whether oral flora contribute to CF airway microbial communities, we compared the normalized average sequence abundance of all OTUs found in 22 paired mouthwash and sputum samples from 9 patients. For each taxon, normalized average sequence abundance values are plotted as a logarithm to the base 10 (log_10_). Red symbols indicate taxa that had significantly different distribution between sample types at a 10% false discovery rate from a parametric t-test in which the 22 samples were treated as independent measurements.

We previously reported quantitative bacterial culture results for all sputum samples included in this study [8]. To validate the pyrosequencing dataset, we compared qualitative culture results (culture positive or culture negative) and relative abundance of sequences for the dominant CF pathogens *Pseudomonas* and *Burkholderia* for each sputum sample (Figure 3). Patient specimens that were culture positive for *P. aeruginosa* had a substantial fraction of all sequences assigned to the genus *Pseudomonas* and few sequences assigned to *Burkholderia.* Similarly, sputum samples that were culture positive for *B. cepacia* complex species had a substantial number of sequences assigned to genus *Burkholderia* and few sequences assigned to genus *Pseudomonas*.

**Figure 9:**
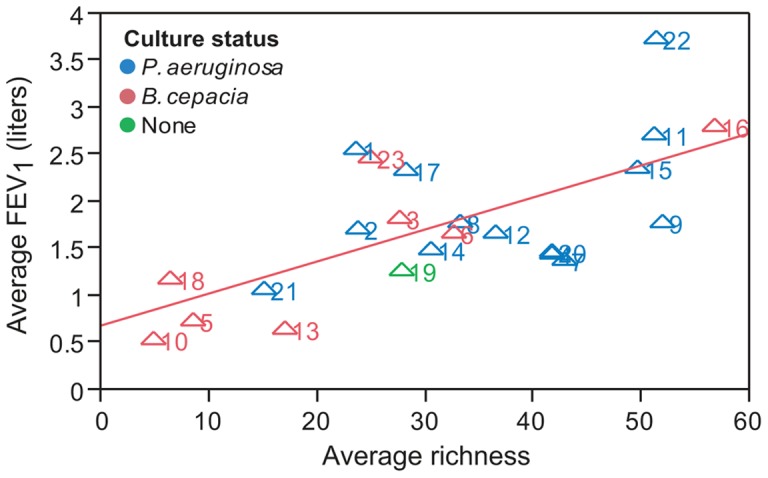
Low species richness in sputum samples is associated with decreased lung function. Shown is a plot of the average FEV_1_ compared to average microbial richness in sputum for each patient. FEV_1_ and microbial richness values were averaged across all timepoints for each patient. Numbers next to each symbol indicate patient ID. Symbols are color-coded based on patient culture status (*P. aeruginosa*, blue; *B. cepacia* complex species, red; culture negative for both, green). Results from linear regression analysis (red line) indicate a significant correlation (r^2^ = 0.42, p = 0.0009).

To determine whether there was quantitative agreement between the two methods, we directly compared total viable counts (TVC) as determined by culture [8] and relative sequence abundance determined by pyrosequencing (Figure 4). Linear regression between TVC and relative sequence abundance were highly significant for both *Pseudomonas* (Figure 4A, black line; r^2^ = 0.71, p<0.001) and *Burkholderia* (Figure 4B, black line; r^2^ = 0.86, p<0.001), demonstrating general agreement between the methods. Despite this agreement, visual inspection of the data suggest that the relationship between TVC and relative sequence abundance may be better characterized by a saturating non-linear model (Figure 4; red lines). This non-linear relationship is presumably due to the inherent difference between relative and absolute abundance measures. For example, when the majority of sequences in a sample are already assigned to a given taxon, a further increase in the absolute abundance of that taxon cannot be reflected by a proportionate increase in the relative sequence abundance. For this reason, changes of more than an order of magnitude for TVC for *P. aeruginosa* and *B. cepacia* are not necessarily reflected in large changes in relative sequence abundance (Figure 4). Regardless of the saturation effect seen for numerically dominant species, this comparison between TVC and pyrosequencing indicates that relative sequence abundance is a reliable means for characterizing microbial community structure and changes in species diversity or richness following antibiotic disturbance.

**Figure 10:**
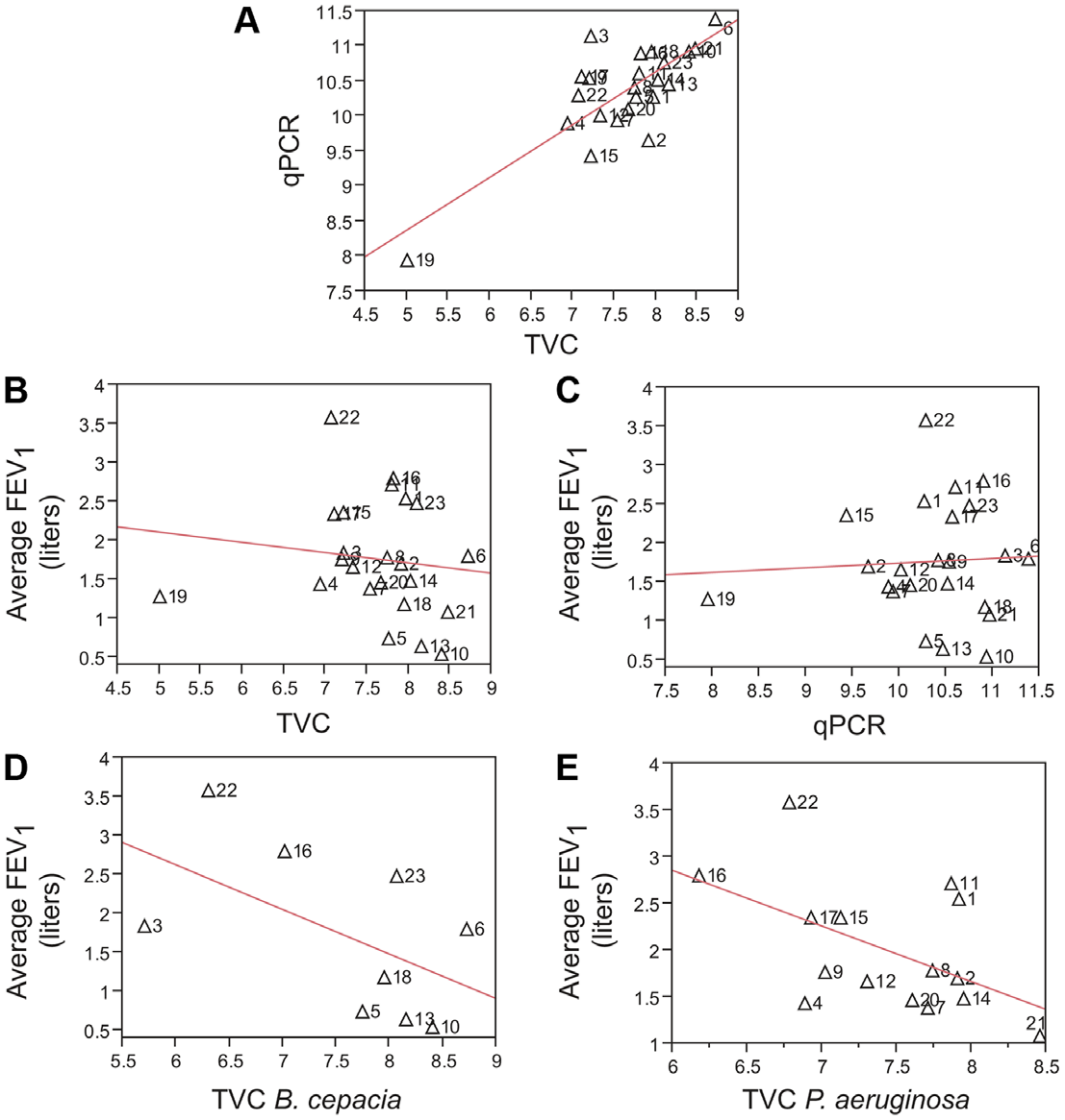
Lung function is not correlated with total bacterial abundance. Measurements of total bacterial abundance in sputum by (a) TVC and qPCR are well correlated (r^2^ = 0.63, p<0.0001; n = 23). FEV_1_ is not correlated with (b) TVC (r^2^ = 0.017, p = 0.55; n = 23) or (c) qPCR (r^2^ = 0.003, p = 0.8; n = 23) and only modestly correlated with TVC from (d) *B. cepacia* complex species (r^2^ = 0.29, p = 0.13; n = 8) and (e) *P. aeruginosa* (r^2^ = 0.26, p = 0.05; n = 14). Measurements for bacterial abundance and FEV_1_ were averaged across timepoints for each of the 23 patients. Labels in each panel indicate patient ID. Lines indicate regression fit by linear least squares. Only TVC values >0 were included for *B. cepacia* and *P. aeruginosa* comparisons. TVC values represent log_10_ of total bacterial colony forming units (CFUs) recovered per gram of sputum. qPCR values represent log_10_ copies of the bacterial 16S rRNA gene detected per gram of sputum.

### Antibiotic treatment reduces low abundance taxa, but has minimal impact on overall CF microbial community structure

While a number of recent studies have described the CF airway microbiome [8], [12], [13], [29]–[31], none have examined the short-term longitudinal impact of exacerbation and antibiotic treatment on a large patient population. The results presented in Figure 3 suggest that there is general stability of the dominant pathogens as measured by sequencing at the onset of exacerbation, following antibiotic treatment (end-of-treatment) and during stable intervals. Notably, samples taken at the end-of-treatment timepoint did not consistently show a lower fraction of sequences assigned to *Pseudomonas* or *Burkholderia* compared to samples taken either at the onset of exacerbation or when patients were clinically stable. To determine whether other less abundant members of the microbial community are affected by antibiotic treatment, we compared the normalized average sequence abundance for all taxa at the onset of exacerbation and at the end-of-treatment with intravenous antibiotics (Figure 5A, Table S6). The relative sequence abundance of the individual OTUs was broadly similar at these two timepoints, indicating that antibiotic treatment did not have a dramatic affect on overall community structure. To determine whether the relative abundance of specific taxa was impacted, we tested the null hypothesis that the distribution of sequence reads for each OTU was the same at the exacerbation and end-of-treatment timepoints (Table S6). At a 10% false discovery rate, we found that the relative sequence abundance of only 7 OTUs (*Gemella*, 2 *Pasteurella* OTUs, 2 *Streptococcus* OTUs, *Oribacterium* and *Neisseria*; indicated by red symbols in Figure 5A) was higher at the beginning of an exacerbation than at the end-of-treatment. With regard to CF airway microbial communities, this result suggests that antibiotic treatment may have a greater impact on a small number of less abundant taxa.

**Figure 11:**
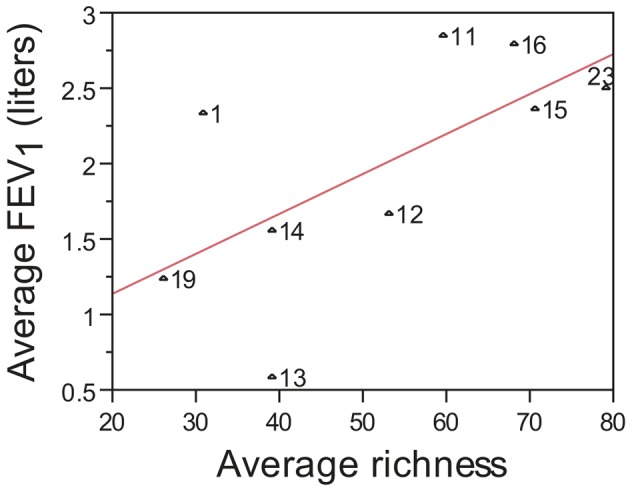
Low species richness in mouthwash samples is associated with decreased lung functions. Shown is a plot of the average FEV_1_ compared to average microbial richness in mouthwash samples for nine patients. FEV_1_ and microbial richness values were averaged across all timepoints for each patient. Numbers next to each symbol indicate patient ID. Results from linear regression analysis (red line) showed a modest correlation with lung function (r^2^ = 0.42, p = 0.057).

Twelve different broad-spectrum antibiotic regimens were used to treat the 26 exacerbations characterized in this study (Table 1). To determine if specific antibiotic combinations had a differential impact on the airway microbiome, we compared the two largest treatment groups (Tobramycin and ceftzidime versus Tobramycin and piperacillin/tazobactam) and found no significant differences in microbial community structure (data not shown). The small sample size for the remaining treatment regimens precluded further statistical comparisons.

To determine whether specific taxa were associated with the onset of exacerbation, we compared the distribution of the sequence reads for each OTU at the beginning of exacerbation and stable timepoints. The pattern of OTUs was highly similar between these timepoints (Figure 5B). The only obvious outlier in this relationship was *Pseudomonas* (Figure 5B, black arrow); however, the difference in the distribution of *Pseudomonas* sequence counts between exacerbation and stable timepoints was not statistically significant (paired t-test; p = 0.13, n = 13).

Visual inspection of Figure 5A shows that there is a higher number of taxa under the identity line (Figure 5A, red line) than over the identity line, suggesting that antibiotic treatment may affect specific taxa in a patient dependent or antibiotic dependent manner that is not apparent in the aggregate data. To further investigate the possibility of patient specific changes in microbial community structure, we calculated richness (the number of taxa present) within each sample. Richness was lower at the end-of-treatment timepoint than at other timepoints (Figure 5C), although this difference did not reach statistical significance (paired t-test, p = 0.06). Despite this trend towards lower richness, the overall similarity of the distributions of taxa between the exacerbation and end-of-treatment timepoints (Figure 5C) demonstrates that antibiotic treatment does not lead to wholesale restructuring of the microbial community.

### Infection types, defined by the dominant CF pathogens *Pseudomonas* and *Burkholderia*, are not associated with distinct microbial communities

A previous study partitioned the CF airway microbiome into core and satellite taxa, as a means to distinguish microbial species that are present in nearly all CF patients from those that display a more variable distribution among patients, respectively [29]. The authors observed a positive correlation between the number of patients that harbored a given taxon and the mean abundance of that taxon [29]. This relationship was also apparent in our patient cohort, where the average number of sequences for each taxon shows a positive linear correlation (Kendall rank correlation; p = 3.12×10^−05^) with the number of samples in which that taxon is present (Figure 6, Table S7). In other words, high abundance in one sample is an indicator that a taxon is likely to be prevalent in the patient cohort.

The two most obvious outliers in this relationship are the OTUs assigned to *Pseudomonas* and *Burkholderia*, which appear to have a very different distribution from other taxa in our study; they are highly abundant when present, but are only detected in largely non-overlapping patient subsets (Figure 3). Previous studies have demonstrated that microbial community structure involves complex interactions between the constituent members and that overall community composition is impacted by numerically dominant species [20], [35]. Since *Burkholderia* and *Pseudomonas* account for the majority of all the sequences observed in this study, and the presence of *Burkholderia* and *Pseudomonas* tends to be mutually exclusive in our dataset, we predicted that the presence of these organisms would have a differential impact on the composition of their respective microbial communities. To determine whether distinct community structures existed within our patient population, we performed a principal coordinates analysis (PCoA) of all sputum samples. Replacing the relative abundance data for each OTU with the first two principal coordinates resulted in a clear separation of the *Pseudomonas* and *Burkholderia* dominated communities (Figure 7A; Table 2). To determine if these differences were reflected in taxa other than *Burkholderia* and *Pseudomonas*, we repeated the PCoA after excluding all *Burkholderia* and *Pseudomonas* sequences. When the *Pseudomonas* and *Burkholderia* sequences were removed, nearly all of the distinctions between the microbial communities were lost (Figure 7B; Table 2). Moreover, when we tested the null hypothesis that a given taxon is differentially represented in *Pseudomonas* or *Burkholderia* dominated communities, only the two *Pseudomonas* OTUs and one *Burkholderia* OTU were detected at a 10% false discovery rate (Table S8). These results suggest that *Pseudomonas* and *Burkholderia* infections occur against a relatively stable background of other members of the microbial community.

### Individuals have distinct airway microbial communities that are stable over time but show subtle transient changes following antibiotic treatment

While the largest difference in CF airway microbial community structure was associated with infection by *Pseudomonas* or *Burkholderia*, PCoA revealed subtle patterns associated with timepoint (Figure 7C–D; Table 3) and individual patients (Figure 7E–F; Table 2). For example, in most patients, end-of-treatment samples tend to separate from the corresponding exacerbation samples, based on both the first and second coordinates of the PCoA (Figure 7C). Although the magnitude of this difference is small, it is observed consistently across patients and we are therefore able to reject the null hypothesis that the exacerbation and end-of-treatment timepoints cluster together (Table 3; coordinate 1, p = 3.15×10^−5^; coordinate 2, p = 0.01). When *Pseudomonas* and *Burkholderia* sequences are removed, the distinction between exacerbation and end-of-treatment timepoints is more pronounced (Table 3; coordinate 1, p = 9.54×10^−6^; coordinate 2, p = 0.002) and it is apparent that both the stable and exacerbation timepoints tend to cluster separately from the end-of-treatment timepoints (Figure 7D, Table 3). As was the case when we examined each OTU individually (Figure 5), PCoA analysis revealed a measurable but transient effect of antibiotics on the end-of-treatment samples, while the exacerbation and stable timepoints were largely indistinguishable. Taken together, these results emphasize the resilience of the microbial community and the short-term effects of antibiotic treatment.

Likewise, samples taken at different timepoints from the same individual tended to cluster more closely over time (Figure 7E–F), and this clustering is apparent whether or not sequences from *Pseudomonas* and *Burkholderia* are included (Table 2). Because each patient was defined by bacterial culture as either *Pseudomonas*- or *Burkholderia*-positive, patient ID is confounded by culture results. To avoid this problem, we analyzed the *Pseudomonas-* and *Burkholderia*-positive patient groups separately. Despite the limited sample size for the two infection types (*Pseudomonas*-positive, n = 14; *Burkholderia*-positive, n = 8), we were able to reject the null hypothesis that individual patients have distinct microbiomes over time, for at least one principle coordinate (Table 2). The finding that individuals have unique microbiomes is consistent with other studies that have shown a distinct individual signature that is maintained over longitudinal sampling [36], [37].

### CF airway and oral microbial communities are highly similar

Numerous studies have noted that the majority of facultative and obligate anaerobes detected in CF respiratory samples are typically associated with the normal human oral microbiota [20], [29], [32], [38]. The use of careful sampling methods and the apparent density of these organisms in sputum strongly suggest that their presence is not explained by sputum contamination with saliva [32], [39]. To examine the overlap between the CF airway and oral microbiota and to better characterize the contribution of oral flora in CF airway infection, we collected mouthwash samples for a subset of 9 CF patients at different timepoints (n = 22 samples) (Table S1). To characterize the oral microbiota, total microbial DNA was extracted from mouthwash samples and was sequenced and analyzed in parallel with the corresponding sputum samples (see Methods). With few exceptions (most notably *Pseudomonas*), the average relative abundance of taxa observed in the mouthwash samples was highly similar to that observed in sputum samples (Figure 8). At a 10% false discovery rate, the Wilcoxon signed-rank test detected only two OTUs (*Pseudomonas* and *Rothia*) that showed a different distribution between mouthwash and sputum; with *Pseudomonas* being more abundant in sputum and *Rothia* more prevalent in mouthwash (Table S9). A parametric t-test recovers six additional OTUs classified as *Granulicatella* (2 OTUs) and *Streptococcus* (four OTUs) that show a higher relative abundance in mouthwash compared to sputum (Table S9). It is important to note, however, that our sample size is small (with mouthwash samples from only 9 individual patients). It is likely that a survey with a larger sample size could detect more taxa that are different between mouthwash and sputum. Within the limits of our sample size, however, our results indicate an underlying CF airway microbiome that is largely reflective of the oral cavity.

### Microbial richness shows significant correlations with patient clinical status

Recent studies using both Sanger sequencing and 454 pyrosequencing methods have found a relationship between reduced species diversity (as measured by richness or Shannon diversity) and reduced respiratory function (as measured by FEV) [29], [40]. We have shown that a two-week course of intravenous antibiotics only modestly diminishes the richness of CF airway microbial communities (Figure 5C), and that this effect is temporary, with richness similar at exacerbation and stable timepoints. As richness is broadly stable across time, we calculated an average richness for each patient by pooling all samples associated with the patient. Average richness, calculated in this way, correlated well (r^2^ = 0.42, p = 0.0009) with FEV_1_, a broad measure of the respiratory function, in our cross-sectional analysis (Figure 9). Our results are, therefore, in agreement with the existing literature [29], [40].

We also determined if total microbial abundance was similarly correlated with FEV_1_. Microbial abundance was previously calculated for all samples in this study by determining TVC by culture and by qPCR targeting the 16S rRNA gene [8]. While these two metrics correlate well with each other (Figure 10A; r^2^ = 0.63, p<0.0001), neither TVC (Figure 10B; r^2^ = 0.017, p = 0.55) or qPCR data (Figure 10C; r^2^ = 0.003, p = 0.8) correlated with FEV_1_. When we only examined TVC associated with *B. cepacia* complex species (Figure 10D; r^2^ = 0.29, p = 0.13) or *P. aeruginosa* (Figure 10E; r^2^ = 0.26, p = 0.05), there was at best a modest correlation with FEV_1_. Therefore, we conclude that microbial richness is a better predictor of FEV_1_ (Figure 9) than measures of total microbial abundance (Figure 10). This is also consistent with a previous study in which total bacterial density remained constant over time despite changes in diversity that were associated with decreased lung function as measured by FEV_1_ [40].

Given the similarity between the oral microbiota and airway microbiota described above, we determined whether reduced FEV_1_ was reflected in reduced diversity in the oral microbiota. Similar to sputum samples, the average richness of microbial species detected in mouthwash samples from the nine CF patients showed a modest correlation with lung function (Figure 11; r^2^ = 0.42, p = 0.057); however, the correlation did not reach the threshold of statistical significance. Given the small sample size, this observation suggests that a larger study of diversity in CF patient mouthwash samples is warranted. Despite the limited number of mouthwash samples examined, these results suggest that CF lung function may correlate with microbial diversity at multiple body sites.

## Discussion

Within the CF patients in our cohort, we observed a largely stable overall structure of the airway microbial community, despite antibiotic treatment and changes in disease status (Figure 5). For the 23 patients included in this study, we observed many species belonging to the genera *Streptococcus, Prevotella*, *Rothia*, *Veillonella*, *Actinomyces*, and *Granulicatella* and a single numerically dominant pathogen, either *Pseudomonas* or *Burkholderia* (Figures 2–3). With few exceptions, the taxa reported here are well represented in Sanger-based sequencing and culture-based CF studies, suggesting that the adult CF microbial community has a distinct signature that can be successfully detected with multiple technologies [8], [9], [12], [29].

Recent studies have emphasized the successional progression and loss of microbial diversity as a function of CF patient age [13], [30]. Our study suggests that at the end point of this succession, poor lung function is associated with a low-diversity community (Figure 9) that it is largely resistant to changes in the face of antibiotic treatment (Figure 5). Similar findings have recently been reported for patients with COPD [41]. Sampling of either expectorated sputum or mouthwash largely reflects the same microbes, with the possible exception of dominant aerobic airway pathogens (Figure 8). Interestingly, while loss of species richness is associated with decreased lung function (Figure 9), we did not find a significant relationship between total abundance of bacteria and decreased lung function (Figure 10). Further, our data generally do not support either intensification or the overgrowth of a particular pathogen or the acquisition of new microbial community members as the cause of acute exacerbations in this patient cohort (Figure 5). This is consistent with a recently published study that observed total bacterial density remaining stable in CF patients even as FEV deteriorated over time [40]. Overall, these findings support the hypothesis that exacerbation represents the spread of infection to previously unaffected airways. Alternatively, exacerbation may be the consequence of further adaptations of the microbial community to the airway environment such as a change in the expression of virulence traits by one or more species within the community. For example, during chronic CF airway infection, *P. aeruginosa* can undergo conversion from a smooth to mucoid colony phenotype, due to over production of the capsular polysaccharide alginate [42]. Mucoid conversion within the *P. aeruginosa* population is associated with a host hyperimmune response and a more rapid decline in lung function [43], [44]. Future studies will be necessary to determine whether exacerbation involves the spread of infection or a change in the behavior of the microbial community.

The finding that microbial community composition did not substantively change with antibiotic treatment is perhaps surprising given that the majority of these patients were treated for two weeks with a combination of broad-spectrum antibiotics (Table 1). While there was a small decrease in richness following antibiotic treatment (Figure 5C), the changes appear to be largely limited to taxa that were already of low relative abundance (Figure 5A). Moreover, change in OTU relative abundance following antibiotic treatments was statistically significant in only a small number of taxa at a 10% false discovery rate (Figure 5A; red symbols). While a larger sample size might detect significant changes in more taxa, it is clear that the most abundant taxa in this cohort (including *Prevotella*, *Actinomyces*, *Granulicatella*, *Streptococcus*, *Burkholderia*, *Veillonella*, *Rothia* and *Pseudomonas*) showed little change in response to antibiotics (Figure 5A). This observation may be due to the fact that antibiotic resistance is widespread throughout many abundant members of the microbiota cultured from the sputum of adult CF patients including *P. aeruginosa* and *B. cepacia* complex species [45]–[47]. As the cost of whole genome sequencing continues drop, it will be interesting to see if the mechanisms of antibiotic resistance in each of these taxa can be located and if the acquisition of resistance elements can be followed longitudinally in individual patients as they age.

One limitation of our results is that they are dependent on a quantitative interpretation of high-throughput pyrosequencing data. While next generation sequencing has generated an unprecedented, detailed view of microbial communities within the human body, current PCR-based technology is prone to saturation effects. Since the CF microbial community can be dominated by a relatively small number of pathogens, these saturation artifacts may be more pronounced than in other polymicrobial diseases. We observed that the agreement between culture and relative sequence abundance is best described by a non-linear model (Figure 4). Moreover, absolute measures of abundance, such as TVC and qPCR targeting the 16S rRNA gene, show a small, but statistically significant, response to antibiotics [8]; whereas, our relative sequence counts largely show stability of microbial community structure in response to antibiotics (Figure 5A). The differences between the two methods may be due to the inherent differences between relative and absolute measurements of abundance. For many of the samples in which *Pseudomonas* or *Burkholderia* were detected, their associated sequences represent more than 50% of the reads in the sample (Figure 3). Under these conditions, further large increases in *Pseudomonas* or *Burkholderia* would not be detected by sequencing as the majority of sequences are already assigned to these dominant pathogens. While pyrosequencing may not detect changes in absolute density of numerically dominant microbial community members, the fact that we detected a similar distribution of taxa in sputum and mouthwash samples (Figure 8), which harbor substantially fewer dominant pathogens, strongly suggests that the observed overall community structure is not a result of saturation artifacts.

In the present study, mouthwash and spontaneous sputum samples had a very similar distribution of taxa, with the exception of the pathogens *Pseudomonas* (Figure 8) and a small number of other taxa (Table S9). This finding is similar to that recently reported for healthy individuals where bacterial communities from the lung displayed compositions indistinguishable from the upper airways [48]. A number of studies have clearly shown that detection in sputum of bacteria, which are members of the normal oral microbiota, is not a result of contamination of the sputum sample during collection [9], [39]. Furthermore, these bacteria are also present in low numbers in the lungs of healthy individuals, healthy smokers and patients with other respiratory diseases characterized by chronic infection such as COPD [41], [48]. Therefore, our findings suggest that the oral cavity could serve as a reservoir for respiratory infection with sputum seeded during small-volume aspiration.

Members of complex microbial communities are known to exhibit both mutualistic and antagonist relationships. Therefore, it is reasonable that we would detect different microbial community organization in patients who had a *Pseudomonas* dominated infection compared to patients with a *Burkholderia* dominated infection. However, our data do not support this hypothesis. When we remove *Pseudomonas* and *Burkholderia* sequences from our data set, the underlying microbial community structures were largely indistinguishable (Figure 7B; Table 2). Moreover, with the exception of *Pseudomonas* and *Burkholderia*, taxa that are present in a large number of patients also tend to be present in the highest abundance (Figure 6). Taken together, these data suggest that *Pseudomonas* and *Burkholderia* infections occur in the context of a highly stable microbial community that is on average, broadly similar across patients. *Pseudomonas* and *Burkholderia* are closely related organisms and it may be that they can play similar ecological roles in the communities in which they exist. Alternatively, the underlying microbial community, which may be continually reseeded by aspiration from the oral cavity, may only support or select for a subset of non-endogenous opportunistic pathogens with a related metabolic capacity.

In summary, we have observed a surprising degree of stability in microbial community composition across sample types and before and after antibiotic treatment of adult patients infected with a range of opportunistic pathogens. Taken together with recent successional studies that have documented higher diversity in children with CF [13], [30], our study supports a model of CF in which a diverse but unstable microbial community associated with children is reduced to a smaller set of highly resistant taxa in adults that are stable across time and space. This model is consistent with the recent observation that lifetime exposure to antibiotics in CF patients is associated with lower FEV and lower microbial diversity [40]. Our data further suggest that the loss of lung function associated with acute exacerbation may reflect intrapulmonary spread of infection to previously unaffected airways rather than a change in microbial density or composition of the airway microbial communities. Future studies that exploit the cultivability of nearly all CF taxa [31] and the increasingly low cost of obtaining whole-genome sequences of cultured taxa may determine the specific molecular mechanisms by which a child' s high-diversity microbiome is replaced in adulthood with a low-diversity highly resistant microbiome. Finally, we conclude that long-term changes in microbial community structure (richness) are likely to be associated with the slow decline in CF lung function over time.

## Methods

### Ethics Statement

This study was conducted with the approval of the Office for Research Ethics Northern Ireland and all study participants provided informed written consent.

### Patient selection and collection of samples

Details of the CF patient cohort were previously published as part of an earlier study in which the airway microbiota was characterized by aerobic and anaerobic bacterial culture, qPCR and by terminal restriction fragment length polymorphism analysis of bacterial 16 S ribosomal RNA genes present in sputum [8]. Exacerbations were defined according to the criteria of Fuchs et al. (1994) and treated with a range of antibiotic [49] (Table 1). In brief, sputum samples were collected at the onset of exacerbation (24 hours before to a maximum of 48 hours after administration of the first dose of intravenous antibiotics) and at the completion of antibiotic treatment (24 hours before to a maximum of 48 hours after last dose of antibiotics). In addition, sputum samples were collected from 13 patients when clinically stable; defined as no change in symptoms, FEV_1_ within 10% of best value in the previous 6 months and no new antibiotics started.

Spontaneously expectorated sputum was collected and transported within 15 minutes to an anaerobic cabinet for processing. All samples were divided with one aliquot immediately processed for culture [8] and a second aliquot frozen and stored at −80°C for subsequent DNA extraction.

Mouthwash samples were collected from a subset of nine patients immediately prior to sputum collection at the time of exacerbation and/or at the end of antibiotic treatment. Patients gargled a 10 ml volume of sterile saline (0.9%) for 30 seconds, and wash fluid was collected in a sputum pot. Samples were frozen and stored at −80°C for subsequent DNA extraction.

DNA was extracted from frozen sputum samples as previously described [8]. Mouthwash samples were thawed and centrifuge at 4°C, at 17,000×g for 10 minutes. Total DNA was isolated from the pelleted material using the FastDNA Spin Kit and FastPrep Instrument (MP Biomedicals) according to the manufacturer' s protocol. Isolated DNA was passed over a Zymo-Spin IV-HRC column (Zymo Research) and the concentration of recovered DNA determined using Quant-iT PicoGreen dsDNA reagent (Invitrogen).

### Amplification and barcoding of bacterial 16S rRNA gene amplicons

The 454 Life Sciences primer B (bold) with a “TC” linker and bacterial 27F primer (underlined) (5′-**GCCTTGCCAGCCCGCTCAG**TCAGAGTTTGATCCTGGCTCAG-3′) and primer A (bold) with an 8 nucleotide barcode (N), “CA” linker, and bacterial primer 338R (underlined) (5′-**GCCTCCCTCGCGCCATCAG**NNNNNNNNCATGCTGCCTCCCGTAGGAGT-3′) were used to target the V1-V2 variable regions of the bacterial 16S rRNA gene. Primer design and barcode sequences were as previously described [50]. PCR amplification of the 16S rRNA gene was performed with barcoded primers as previously described, using AmpliTaq Gold 2x Mastermix (Applied Biosystems) [51]. For each patient sample, one or more PCR reactions were pooled to achieve a minimum of 50 ng of amplicon. All reactions were cleaned and concentrated with Montage PCR Clean-Up Kit (Millipore), and DNA concentrations determined with Quant-iT PicoGreen dsDNA Assay Kit from Molecular Probes (Life Technologies) on a Synergy HT fluorescent plate reader from BioTek Instruments. Volumes representing equal mass of DNA were pooled into a single tube and concentrated using a Qiaquick PCR Purification Kit (Qiagen Inc.) DNA was briefly subjected to SpeedVac (Thermo Scientific) with heating to ensure all residual ethanol was removed.

### Pyrosequencing and sequence quality control filtering

Pyrosequences were generated using the 454-FLX chemistry platform (Roche) on two separate sequencing plates at the University of North Carolina High Throughput Sequencing Facility. Sequences not having an exact match to a 5′ primer were removed from the dataset. The program Lucy (v. 1.20) [52] was used to end-trim the remaining sequences to a quality score (Q-score) of 27 (p = 0.002) as previously recommended [53]. Additional trimmed sequence were removed from the dataset based on the following criteria: (i) the trimmed sequence contained any N' s; (ii) the trimmed sequence was shorter than 180 base pairs or longer than 470 base pairs or (iii) the 5′ region was identified as low-quality by Lucy in the untrimmed sequence (below a Q-score of 27). A total of 405,888 sequences met these criteria and were used for subsequent analysis in version 2 of AbundantOTU [27]. Using default parameters (which cluster sequences with an average percent identity of 97%), AbundantOTU clustered 97.8% of the 405,888 sequences into OTUs. Sequences that were not incorporated into an OTU were removed from further analysis. The consensus sequences for each OTU were analyzed with the Broad Institute' s implementation of ChimeraSlayer [54]. 34 chimeric OTUs were detected and removed from downstream analysis. The program UCHIME [55] was run on the remaining consensus sequences with the Gold database as reference. UCHIME reported no chimeras among these 177 consensus sequences, which were used for further analysis.

To remove non-microbial sequences, we mapped each consensus sequence to version 10.22 of the RDP database [56] using the 64 bit version 2.2.18 of NCBI' s BLAST. All but two consensus sequences (Consensus172 and Consensus202) matched to a known microbial sequence within the RDP database with an alignment length of at least 183 base pairs and a percent identity of at least 95.8%. Comparison of the consensus sequences with the same version of BLAST to version 104 of the SILVA reference tree (http://www.arb-silva.de/) yielded similar results; all but the same two consensus sequences matched to a known microbial sequence in the SILVA database with an alignment length of at least 113 base pairs and a percent identity of 95.4%. Using NCBI BLAST (http://blast.ncbi.nlm.nih.gov/Blast.cgi) against the NR database on the two consensus sequences that did not match either RDP or SILVA revealed that one of the consensus sequences was a human contaminant and the other had no matching sequences in the NR database. These two OTUs were therefore removed from downstream analysis resulting in a total of 175 consensus OTUs generated from the 85 samples examined in this study (63 sputum, 22 mouthwash). Figure 1 shows the results of using the program align.seqs in the package Mothur [57] to map the sputum-derived non-chimeric microbial consensus sequences to version 104 of the SILVA database. To reduce the memory requirements of the align.seqs method, only the SILVA sequences that were detected by the BLASTN search of the consensus sequences were included in the “template” file for the align.seqs search.

Table S1 shows the number of sequences for each sputum sample that passed all QC steps and were included in a non-chimeric OTU for which the consensus sequence mapped to a known microbial sequence. Because all of the consensus sequences for each of the OTUs in our analysis pool had an excellent (>95%) match by BLAST to the SILVA reference tree and all but two of the consensus sequences had a >95% match across the global alignment produced by align.seqs (Figure 1), the SILVA reference tree was used as a basis for creation of the phylogenetic visualization in Figure 2. Within the SILVA reference tree, each consensus sequence was substituted for the node on the tree that had the closest match (as determined by the align.seqs program in Mothur). All other nodes were removed from the tree using custom Java code and the function deleteSubtree from the function org.forester.phylogeny Phylogeny within the Archaeopteryx (http://www.phylosoft.org/archaeopteryx/) source code. Each reference sequence in the SILVA database was classified with v. 2.1 of the standalone version of the RDP classifier and the most refined classification at a confidence score of 80% was used to label each leaf in Figure 2. This was accomplished with custom Java code to produce a PhyloXML file, which was visualized with Archaeopteryx.

### Sample normalization

In our dataset, the number of sequences per sample ranged from 2,292 to 7,212 with an average of 4,300.1 sequences per sample. To correct for variation in total sequence counts between samples, the abundance of each OTU in a given sample was standardized by calculating the logged sequence abundance using the following formula:
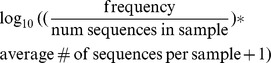



The logarithm was used to lessen the influence of more dominant OTUs. In order to take the log of zero, a pseudo-count (one sequence) is added to each sample for each taxon. In order to minimize the effect of this pseudo-count, all samples are normalized to the average number of sequences per sample before the addition of the pseudo-count.

### Statistical tests and sample size

We obtained sequences for paired exacerbation and end of treatment timepoints for 21 patients (Table S1). For two additional patients, we were unable to obtained sequences for the exacerbation sample (patient 22) or the end of treatment sample (patient 12), because sample derived total DNA was limited. For 3 patients (patient 1, patient 11 and patient 19), we generated sequences for second set of paired exacerbation and end of treatment timepoints. Finally, for 13 patients (patients 1, 2, 4, 7, 8, 9, 11, 13, 14, 17, 19, 21 and 23), we collected sequences for a stable timepoint taken either before or after the exacerbation event.

For paired t-tests comparing exacerbation to end of treatment timepoints (Figure 5C, Table 3), we interrogated 21 paired samples (the second set of paired exacerbation and end of treatment samples for patients 1, 11 and 19 were not included to avoid violating the assumption of independence). For paired t-tests comparing exacerbation and stable timepoints (Figure 5C, Table 3), we considered the earliest exacerbation timepoint and the stable timepoint to yield a samples size of 13.

Statistical tests were performed using the program JMP (http://www.jmp.com/). PCoAs were performed in the package Mothur (using the program pcoa) based on Bray-Curtis dissimilarity defined as:
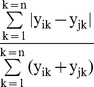
Where y_ik_ and y_jk_ are the log-transformed and normalized values for taxa k in samples i and j respectively and n is the number of taxa in all samples. Only taxa that had at least 10 total sequences across all samples were considered in the Bray-Curtis distance measurement.

## Supporting Information

Table S1 **Sample summary.** Description of the 85 samples used in this study (63 sputum, 22 mouthwash).
(TXT)

Table S2 **Rank abundance of sputum OUTs.** List of the 169 OTUs that were found in the CF sputum sample set, ranked by sequence abundance.
(TXT)

Table S3 **Sequence counts for each OTU by sample.** Spreadsheet of the 175 OTUs and their sequence counts for all 85 samples included in this study.
(TXT)

Table S4 **RDP classification for all OTUs.** RDP classification string for 175 OTUs identified in this study.
(TXT)

Table S5 **Consensus nucleotide sequence of all OTUs.** Consensus sequences for the 175 OTUs identified in this study in FASTA format.
(TXT)

Table S6 **Analyses of OTU distribution by timepoint.** Values for all taxa observed in sputum samples in this study are given as the average log transformed normalized number of sequences for each timepoint (Exacerbation Average, EOT Average, Stable Average). The columns “Exacerbation Vs EOT p Value” and “Exacerbation Vs Stable p Value” shows the results of a null hypothesis test (evaluated with a paired t-test) that the distribution of each taxon is the same between timepoints. The column “Corrected P value” show the Benjamani-Hochberg correction equal to N * p/rank where N is the number of rows in the spreadsheet, p is the p-value for this row and rank is the row number. Data was used to generate Figure 5C. Table includes only the 163 OTUs for the sputum sample set considered in these analyses (exacerbation, n = 21; end-of-treatment, n = 21; stable, n = 13). The second exacerbation and end-of-treatment samples for patients 1, 11 and 19 were not included to avoid violating the assumption of independence.
(TXT)

Table S7 **OTU abundance and prevalence.** Values for the average log_10_ normalized number of sequences for each taxon (considering only the samples where the taxa was present) and the number of samples in which the taxon is present. Data was used to generate Figure 6.
(TXT)

Table S8 **Taxa associated with** ***Pseudomonas*** **or** ***Burkholderia*** **culture status**. The results of paired t-tests evaluating the null hypothesis that each taxon has the same distribution in samples identified as *Pseudomonas* or *Burkholderia* by clinical culture. For each patient, the average for each OTU was taken across all timepoints. The Benjamani-Hochberg method was used to generate corrected p values. Table contains the 161 OTUs derived from the sample set used in this analysis.
(TXT)

Table S9 **Comparison of OTUs in paired sputum and mouthwash samples.** The results of parametric t-tests and Wilcoxon tests evaluating the hypothesis that for each taxon, the average distribution in mouthwash samples is the same as in paired sputum samples. For all 9 patients, all samples were averaged across all timepoints. Twenty-two paired samples from 9 patients were treated as independent observations for the calculation of p-values. Data was used to generate Figure 8. Table contains 161 OTUs associated with the paired samples that were analyzed.
(TXT)
